# Clinical utility of the Snaith-Hamilton-Pleasure scale in the Chinese settings

**DOI:** 10.1186/1471-244X-12-184

**Published:** 2012-10-31

**Authors:** Wen-hua Liu, Ling-zhi Wang, Yu-hua Zhu, Min-hui Li, Raymond CK Chan

**Affiliations:** 1 Faculty of Health Management, Guangzhou Medical University, Guangzhou, China; 2 Department of Clinical Psychology and Rehabilitation, Guangzhou Psychiatric Hospital, Guangzhou, China; 3 Neuropsychology and Applied Cognitive Neuroscience Laboratory, Key Laboratory of Mental Health, Institute of Psychology, Chinese Academy of Sciences, 4A Datun Road, Beijing, 100101, China

**Keywords:** Anhedonia, Depression, SHAPS, Assessment, Validation, Chinese

## Abstract

**Background:**

The Snaith-Hamilton-Pleasure-Scale (SHAPS) is a self-reported scale evaluating anhedonia for neuropsychiatric disorders. It has demonstrated with impressive psychometric properties and advantages in its applicability over other similar instruments. However, very few studies have been conducted to examine the clinical utility of the SHAPS in the context of Chinese settings. The current study aimed to examine the clinical utility of the translated version of the SHAPS in the Chinese clinical settings.

**Methods:**

A Chinese version of SHAPS was administered to 336 college students to examine the internal consistency and test-retest reliability at a 4-week interval. Moreover, the translated SHAPS was also administered to 141 patients with major depression, 72 patients with schizophrenia, and 72 healthy controls to examine its clinical discrimination.

**Results:**

The internal consistency of the SHAPS for the non-clinical sample and test-retest reliability at a 4- week interval were 0.85 and 0.64, respectively. Moreover, the SHAPS also showed an excellent internal consistency (alpha was 0.93) and a one-factor solution with the first factor accounted for 51.53% of the variance in the clinical psychiatric samples. ANOVA of the SHAPS total score indicated that the patients with depression scored significantly more anhedonia than the patients with schizophrenia and healthy controls (p<0.001), and the patients with schizophrenia scored significantly more anhedonia than the healthy controls (P<0.02).

**Conclusions:**

These findings suggest that the Chinese version of the SHAPS is a useful and promising instrument in assessing anhedonia for clinical patients and non-clinical individuals in the Chinese settings.

## Background

Anhedonia refers to the inability to experience pleasure or a related reduction of ability to react to pleasurable stimuli, which has been considered to be one of the key features for major depression and schizophrenia [1]. Individuals with anhedonia generally display reduced reward sensitivity [2] or decreased physical response (e.g., heart rate and facial expressions) to emotion-eliciting pictures or words [3]. Although laboratory measures have the advantage of providing an objective, quantifiable measure of hedonic capacity, the highly constraint laboratory-based environment has its limitations to everyday life situation which is more complex and full of distracters. Therefore, the use of self-reported checklist is still very useful for clinical assessment. The most commonly used scales in depression research are the Snaith-Hamilton Pleasure Scale (SHAPS) [4], the Fawcett-Clark Pleasure Capacity Scale (FCPS) [5], and the Revised Chapman Physical Anhedonia Scale (CPAS) [6]. Leventhal et al. (2006) have examined the psychometric properties of these three self-reported scales. Confirmatory factor analysis demonstrated that both the SHAPS and FCPS, but not the CPAS, defined a hedonic capacity construct which was different from depression and anxiety, and hedonic capacity was largely defined by the SHAPS with the highest factor loading on the hedonic capacity variable among the three scales [7]. In addition, the SHAPS also shows more merits over the other similar scales for its brevity and less subject to cultural, sex and age biases [4].

The SHAPS is a 14-item checklist covering four domains of hedonic experience, namely interest/pastimes, social interaction, sensory experience, and food/drink. It has been widely used and translated to different versions including French [8], German [9], Dutch [10], American [11], Japanese [12] and Italian [13]. Impressive psychometric properties and clinical utility have been demonstrated in these versions [8-10,13]. However, to our knowledge, most of the previous studies have been limited to western samples. Very few of them have been done in the non-western samples.

The purpose of the present study was to validate the translated version of the SHAPS in the Chinese settings. In particular, we aimed to examine the internal consistency, convergent and divergent validity, and a test-retest reliability of the SHAPS in a non-clinical sample. Moreover, we further examined its clinical utility and discrimination in patients with depression and schizophrenia.

## Methods

### Participants

For validating the SHAPS in the non-clinical sample, 336 college students (57.1% females) were recruited from two universities located in Guangzhou and Beijing in China. The mean age of the sample was 19.43 years (SD=1.02; range 18–22 years). Fifty-six participants (22 males and 34 females) were randomly invited to complete the SHAPS 4 weeks later. The mean age of these re-test participants was 19.7 years (SD=0.87).

Moreover, 141 out-patients with depression and 72 in-patients with schizophrenia, and 72 healthy controls were recruited to examine the clinical validation and discrimination of this scale. All patients were recruited from Guangzhou Psychiatric Hospital. Clinical diagnoses were determined based on the Structured Clinical Interview for DSM-IV [1] by the experienced psychiatrists (LZW,YHZ,MHL). Patients with any other concurrent Axis I disorders were excluded in this study. The healthy controls were recruited from the local community and were screened by the experienced psychiatrists to ascertain the healthy controls did not have any neuropsychiatric symptoms. There was no significant difference among these three groups on gender, age and the level of education. Tables 1 and 2 summarize the demographic information for the current samples.

**Table 1:** The demographic and emotional information of the non-clinical sample

	**Participants (n=336)**
Gender (M/F)	144/192
Age (years)	19.43±1.02
Education (years)	13.14±1.11
SHAPS (14–56)	19.92±4.52
BDI (0–21)	5.66±5.61
BAI(0–21)	6.05±6.06
PANAS-PA(10–50)	29.57±6.76
PANAS-NA(10–50)	18.36±5.92
SWLS(7–35)	20.42±5.93
TEPS-ANT (11–66)	45.14±5.66
TEPS-CON (9–54)	38.43±5.87
TEPS total score(20–120)	84.89±11.95

Data are presented as n or mean ± SD.

Notes: SHAPS is the Snaith–Hamilton Pleasure Scale, SWLS is the Satisfaction with Life Scale, PANAS is the Positive and Negative Affect Scales, PANAS-PA is the PANAS Positive affect subscale, PANAS-NA is the PANAS Negative affect subscale, BDI is the Beck Depression Inventory, BAI is the Beck Anxiety Inventory, TEPS is the Temporal Experience of Pleasure Scale, TEPS-ANT is the TEPS anticipatory pleasure subscale, TEPS-CON is the TEPS consummatory pleasure subscale.

**Table 2:** Characteristics of the patients with depression, schizophrenia, and healthy controls

	**Healthy controls (n=72)**	**Patients with schizophrenia (n=72)**	**Patients with depression (n=141)**
Gender (M/F)	29/43	36/36	63/78
Age (years)	30.88±10.45	33.12 ±11.09	30.84±10.41
Education (years)	12.52±2.29	12.04±3.17	11.88±3.13
BDI (0–63)	2.91±2.90	9,90±10.18	20.51±12.81
SHAPS (14–56)	21.50±5.17	24.25±7.09	28.15±7.74
TEPS-ANT (11–66)	45.29±7.34	40.34±9.20	40.77±7.60
TEPS-CON (9–54)	39.18±7.59	33.11±7.94	32.26±7.54
TEPS total score	84.47±12.46	73.45±15.92	73.04±13.97

Data are presented as n or mean ± SD.

### Measure of anhedonia severity

The Snaith-Hamilton Pleasure Scale (SHAPS) [4] was used to assess the anhedonia for the current study. It is a 14-item checklist to assess individual’s pleasure experience in the “last few days”. It was rated on a 4-point Likert scale from definitely agree to definitely disagree instead of the original dichotomous scoring (agree and disagree score 0 and 1) proposed by Snaith et al. [4]. The total score ranges from 14 to 56 for the Chinese version of the SHAPS. This scoring method has also been adopted by the validation studies of the Japanese [12] and Dutch [10] versions of the SHAPS. The advantage of this method is that it allows us to yield a more dispersion of the data to calculate the internal consistency, construct validity, and convergent correlations with other scales.

Permission was obtained from the original author of the scale for translation and research purposes. The translation underwent a 2-stage translation process. The first author of this manuscript and an independent professional translator translated the SHAPS into Chinese. The translated version was then back translated into English by two postdoctoral students who did not get involved in the original validation process. The final translation was examined by an expert panel consisting of clinicians and researchers to ascertain the items are culturally relevant and can be correctly understood by the Chinese participants.

### Measures of convergent validity

The Temporal Experience of Pleasure Scale (TEPS) [14] was used as a concurrent measure of anhedonia to evaluate different components of the long-term experience of pleasure, namely the anticipatory and consummatory among the participants. The original English version of TEPS has good internal consistency and test-retest reliability [14]. The current study used a 20-item Chinese version that was modified from the original English version (18 items) to consider cultural differences. The Chinese version of TEPS has been proved to possess adequate reliability in previous studies [15-17]. The Cronbach’s alphas for TEPS-ANT (anticipatory pleasure) and TEPS-CON (consummatory pleasure) in the current sample were 0.66 and 0.73, respectively.

The Beck Depression Inventory (BDI) [18], Positive and Negative Affect Scales (PANAS) [19] and Satisfaction with Life Scale (SWLS) [20] were chosen because the absence of hedonic tone may influence individuals’ emotional state and feeling about life. The Beck Depression Inventory (BDI) is a 21-item scale that evaluates the severity of depression. The Chinese version of BDI has excellent psychometric properties [21] and the alpha in the current sample for BDI was 0.83.

The Positive and Negative Affect Scales (PANAS) [19] include 20 items that assess positive and negative affect. The psychometric properties of the Chinese version of the PANAS scales are good [22]. In the present sample, the Cronbach’s alphas for positive affect and negative affect were 0.84 and 0.85, respectively.

The Satisfaction with Life Scale (SWLS) [20] is a 5-item scale to assess global life satisfaction. The Chinese scale has good psychometric properties[23] and the alpha in the current sample was 0.77.

### Measure of divergent validity

The Beck Anxiety Inventory (BAI) [24] was used to evaluate the divergent validity of the SHAPS. The Beck Anxiety Inventory (BAI) is a 21-item scale that assesses anxiety symptoms. The Chinese version of the scale has been validated in Chinese samples [21]. The alpha in the current sample for BAI was 0.87.

### Procedure

In the validity study of the SHAPS in non-clinical sample, all the questionnaires were administered in group format to the college students. Fifty-six of them were randomly invited to complete the SHAPS 4 weeks later. For the clinical samples, patients with depression and schizophrenia and healthy controls were invited to complete the questionnaires on a one-to-one basis in the hospital. The study was approved by the ethics committees of the Guangzhou Medical University and informed consent was obtained from all of the participants.

### Data analysis

Cronbach's α was used to assess the internal consistency of the SHAPS. Principal component analysis, with varimax rotation, was performed to evaluate the factor structure of the SHAPS. Convergent and divergent validity were assessed by evaluating the Pearson correlations between the SHAPS total score and the scores of the BDI, BAI, SWLS, TEPS, and PANAS. Finally, ANOVA was performed to examine the prevalence of anhedonic symptoms between patients with depression and schizophrenia, and healthy controls, and post hoc LSD tests were performed in cases of significant ANOVA effects.

## Results

### Validation in non-clinical sample

The internal consistency (Cronbach's alpha) of the SHAPS was 0.85. The mean item-total correlation was 0.50 (ranging from 0.37 to 0.59). A principal component analysis showed a three-factor solution. The eigenvalues of the first three factors were 4.8, 1.2, and 1.0, respectively. Further inspection of the factor scores explained 34.5% of the total variance with the item load high (mean=0.61) on the first factor.

Table 3 displays the correlations between the SHAPS and the other measures. The SHAPS was inversely correlated with the TEPS anticipatory pleasure (r=−0.47), TEPS consummatory pleasure (r=−0.54), SWLS (r=−0.30) and PANAS positive affect (r=−0.34), and was positively related with the PANAS negative affect (r=0.12) and BDI (r=0.14). In addition, no relation emerged between the SHAPS and BAI (r=0.06). The scores of the SHAPS were not associated with age (r=0.10). However, gender difference existed with females reporting more anhedonic symptoms than males (t(334)=4.75, p<0.001).

**Table 3:** The Pearson correlation coefficients between the SHAPS and other scales in the non-clinical sample

	**SHAPS**	**TEPS_ANT**	**TEPS_CON**	**PANAS PA**	**PANAS NA**	**SWLS**	**BDI**
TEPS_ANT	−0.47(**)			.			
TEPS_CON	−0.54(**)	0.57(**)					
PANAS PA	−0.34(**)	0.26(**)	0.28(**)				
PANAS NA	0.12(*)	0.07	−0.02	−0.10			
SWLS	−0.30(**)	0.16(**)	0.24(**)	0.37(**)	−0.20(**)		
BDI	0.14(**)	−0.12(*)	−0.05	−0.33(**)	0.50(**)	−0.33(**)	
BAI	0.06	−0.04	−0.03	−0.08	0.58(**)	−0.22(**)	0.50(**)

Notes. N=336. *p<0.01, **p<0.001.

### Test–retest reliability

The mean scores of the SHAPS on the first (20.88; SD=4.32) and second (20.16; SD=4.36) test were not significantly different (t(55)=1.45, p>0.05). The intraclass correlation coefficient (ICC) between test and retest on the SHAPS was satisfactory (r=0.64, p<0.001), suggesting adequate test-retest reliability.

### Clinical discrimination of the SHAPS between patients with depression, schizophrenia and healthy controls

One-way ANOVA indicated a significant between groups effect (F(2,282)=22.94, p<0.001) for the SHAPS scores. Post-hoc tests showed that the patients with depression (28.15±7.74) had higher SHAPS scores than the healthy controls (21.50±5.17) and the patients with schizophrenia (24.25±7.09) (all P<0.001). The patients with schizophrenia had higher SHAPS scores than the healthy controls (P<0.02).

Further analysis indicated there was an excellent internal consistency and convergence of the SHAPS in the current clinical samples. The Cronbach's alpha of the SHAPS for the total clinical samples (patients with depression and schizophrenia) was 0.93. The mean item-total correlation was 0.66 (ranging from 0.55 to 0.73). The Cronbach's alphas of the SHAPS in the patients with schizophrenia and depression were 0.91 and 0.93, respectively. A principal factor analysis yielded a one-factor solution for the SHAPS (eigenvalues of the first two initial factors were 7.21 and 0.98, respectively). All items loaded reasonably high (mean=0.72) on the first principal factor, which explained 51.53% of the total variance. Significant correlation was found between the SHAPS and BDI (r=0.45, P<0.001). Moreover, the SHAPS was inversely correlated with both the anticipatory (r=−0.45, P<0.001) and consummatory pleasure subscales (r=−0.50, P<0.001) of TEPS.

## Discussion

This study examined the reliability and validity of the SHAPS in both clinical and non-clinical samples. The SHAPS had good internal consistency (α=0.85) and the principal component analysis yielded a three-factor solution in the non-clinical sample, which are in line with the earlier findings in normal college students population [10]. The test-retest reliability at a 4- week interval (ICC=0.64) was similar to that reported by a previous Dutch study (ICC=0.70) [10]. Because the SHAPS is generally used to measure state anhedonia in neuropsychiatric patients, to some extent, the prevalence of choosing negative items in the non-clinical population is expected to be low and scores on the SHAPS may change over time. Therefore, the test-retest reliability of the SHAPS was not excellent, but at an acceptable level. Moreover, consistent with prior studies in patients with depression [8,10,11] and patients diagnosed with Parkinson’s disease [12,13], findings of this study showed that the SHAPS had excellent internal consistency and adequate construct validity, as indicated by high Cronbach's alpha value (α=0.93), high item-total correlations (mean=0.66) and a one-factor solution with the first factor accounts for 51.53% of the variance in the clinical psychiatric samples. The findings of different dimensionality of the SHAPS in the non-clinical and clinical samples suggest that this scale seems to be more suitable for assessing the hedonic capacity in clinical patients, which is consistent with the supposition that the SHAPS was developed as a unidimensional instrument for clinical research utility [4].

Convergent and discriminant validity were assessed by evaluating the correlations between the SHAPS total score and other scales scores (TEPS, BDI, PANAS, SWLS and BAI). The results demonstrated that the individuals with higher SHAPS scores displayed more deficits in trait measures of anticipatory (r=−0.47) and consummatory pleasure (r=−0.54), which indicated a high relationship between state and trait measures of hedonic capacity. Anhedonia measured by the SHAPS was inversely correlated with positive affect (r=−0.34) and mildly correlated with negative affect (r=0.12). Moreover, the SHAPS scores had a low correlation with depression (r=0.14) and were not related with anxiety (r=0.06). Anhedonia is defined as the absence of pleasurable feeling, and not the mere presence of aversive emotions (such as negative affect, depression or anxiety). Consistent with prior studies [7,10], these findings suggest that the validity of the SHAPS seems to be a pure measure of anhedonia scale, which may tap a related but distinct construct from depression and negative affect [7,10], and has unrelated psychometric properties as compared with anxiety[7]. Finally, the SHAPS was also inversely correlated with general life satisfaction (r=−0.30), suggesting that anhedonia might decrease the feeling of life.

On the other hand, correlation analyses also showed that the patients with depression demonstrated more anhedonic symptoms than the patients with schizophrenia and non-clinical individuals. Moreover, the difference in the SHAPS scores for the patients with schizophrenia and healthy controls was also significant. Although anhedonia is an important symptom of the schizophrenia, there is actually very little research that has examined anhedonic symptoms measured by the SHAPS in patients with schizophrenia. Studies suggest that patients with schizophrenia, especially for those patients associated with "negative" or "deficit" syndromes [25] and severity of disorganization symptoms [25,26], exhibit marked deficits in the hedonic tone when assessed using "trait" measures of affect, but not in their state experience of pleasant stimuli [27]. Anhedonia in patients with schizophrenia did not show consistent correlation with depressive symptoms [28,29]. This may partly explain that the patients with schizophrenia had lower SHAPS scores than the patients with depression in this study. Thus, it seems that the SHAPS scale can be used to discriminate patients with depression and schizophrenia from healthy controls.

Furthermore, this study also examined the relationship of the SHAPS with the demographic characteristics of the participants. We found that the SHAPS was not associated with age and level of education, but was influenced by gender. Females in Chinese sample reported more anhedonic symptoms than males. These findings suggest there is gender difference in the current Chinese sample in responding to pleasant stimuli. Further studies are needed to investigate the possible cross-cultural differences of hedonic capacity between western and non-western samples.

Limitations of the present study include the fact that the sensitivity of self-report anhedonia questionnaire has not yet been evaluated in the large clinical samples, additional studies will also be needed to assess whether the SHAPS scale may be useful for distinguishing patients with different psychopathological disorders. In addition, because inclusive scope for evaluating convergent and discriminant validity of the SHAPS in this study was relatively limited, further examination of the relationships between the SHAPS and other scales are required.

## Conclusions

To summarize, the SHAPS is an easy, simple to administer, reliable and valid questionnaire for assessing anhedonia in both patient and non-patient populations. These current findings facilitate further research to address the role of anhedonia in patients with depression and schizophrenia.

## Abbreviations

DSM- IV: Diagnostic and Statistical Manual of Mental Disorders criteria fourth edition; SHAPS: Snaith–Hamilton Pleasure Scale; SWLS: Satisfaction with Life Scale; PANAS: Positive and Negative Affect Scales; PANAS-PA: Positive Affect Subscale; PANAS-NA: Negative Affect Subscale; BDI: Beck Depression Inventory; BAI: Beck Anxiety Inventory; TEPS: Temporal Experience of Pleasure Scale; TEPS-ANT: TEPS Anticipatory Pleasure; TEPS-CON: TEPS Consummatory Pleasure.

## Competing interests

The authors declare that they have no competing interests.

## Authors’ contributions

WHL designed the study, collected and analyzed the data, and wrote the first draft of the paper. LZW, YHZ, MHL collected the data and assisted data analyses. RCKC conceived and designed the study, and wrote the first draft of the paper. All authors read and approved the final manuscript.

## Pre-publication history

The pre-publication history for this paper can be accessed here:

http://www.biomedcentral.com/1471-244X/12/184/prepub
